# New pencil graphite electrodes for potentiometric determination of fexofenadine hydrochloride and montelukast sodium in their pure, synthetic mixtures, and combined dosage form

**DOI:** 10.1186/s13065-020-00716-z

**Published:** 2020-10-19

**Authors:** Dania Nashed, Imad Noureldin, Amir Alhaj Sakur

**Affiliations:** Analytical and Food Chemistry Dept., Faculty of Pharmacy, Aleppo University, Aleppo, Syria

**Keywords:** Graphite sensors, Potentiometric, Fexofenadine hydrochloride, Montelukast sodium, Molybdate ammonium, Cobalt nitrate

## Abstract

This paper introduces the first electrochemical approach for the determination of Fexofenadine hydrochloride and Montelukast sodium as a combined form by constructing three new graphite electrodes coated with a polymeric membrane. The first electrode was constructed using ammonium molybdate reagent as an ion pair with fexofenadine cation for the determination of Fexofenadine drug, the second electrode was constructed using cobalt nitrate as an ion pair with montelukast anion for the determination of Montelukast drug, the third electrode was prepared by incorporating the two previously mentioned ion pairs in the same graphite sensor, which makes this sensor sensitive to each Fexofenadine and Montelukast drug. The coating material was a polymeric film comprises of Poly Vinyl Chloride (PVC), Di-butyl phthalate as a plasticizer (DBP), ion pairs of drugs with previously mentioned reagents. The electrodes showed a Nernstian response with a mean calibration graph slopes of [59.227, 28.430, (59.048, 28,643)] mv.decade^−1^ for the three pencil electrodes respectively, with detection limits 0.025 μM for Fexofenadine and 0.019 μM for Montelukast drug which makes this method outperforms the reported method for the determination of this combination. The electrodes work effectively over pH range (2–4.5) for Fexofenadine hydrochloride and (5–9.5) for Montelukast sodium. The influence of the proposed interfering species was negligible as shown by selectivity coefficient values. The effectiveness of the electrodes continued in a period of time (45–69) days. The suggested sensors demonstrated useful analytical features for the determination of both drugs in bulk powder, in laboratory prepared mixtures and their combined dosage form. We have validated the method following ICH protocol, and we have reached very significant results in terms of the linearity, accuracy, selectivity, and precision of the method.

## Introduction

**Fig. 1:**
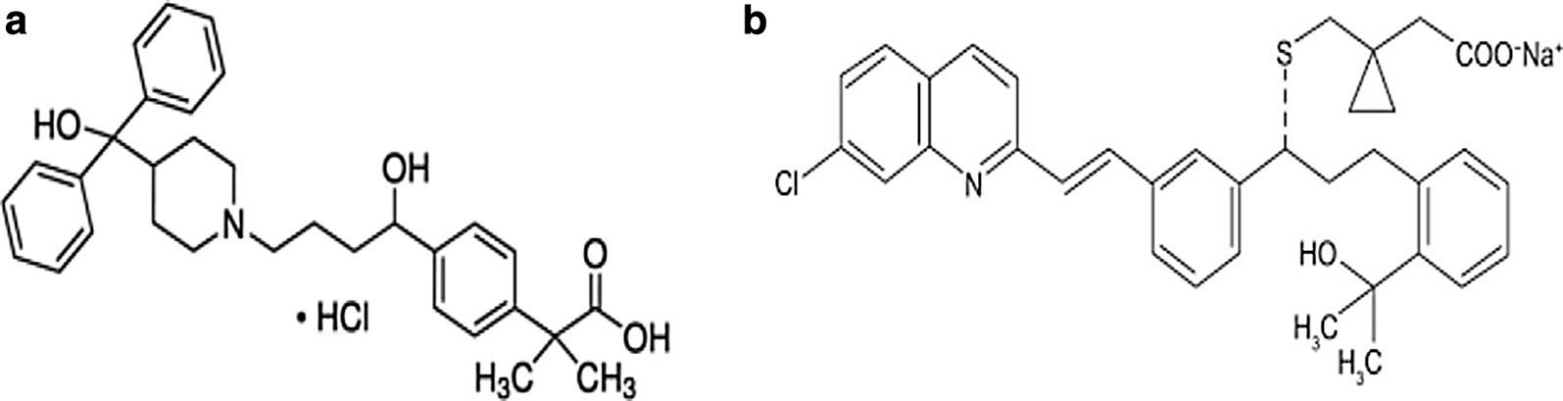
Chemical structure of (**a**) fexofenadine hydrochloride (**b**) Montelukast sodium

Fexofenadine hydrochloride (FEX.HCl) Fig. 1a, is a selective antagonist for histamine H1- receptor, it is an effective metabolite of terfenadine. Its chemical name is (*RS*)2-[4 [1-Hydroxy-4[4-(hydroxy-diphenyl-methyl)-piperidyl]butyl]phenyl]-2-methylpropanoic acid [1], Fexofenadine described as a second or third-generation antihistamine, on 25 February 2000 FDA approved the utilization of Fexofenadine for the handling of periodical allergic rhinitis and chronic urticaria. It restrains the exacerbation of coryza and urticaria and reduces the severity of the signs associated with those conditions such as sneezing, runny nose, irritating eyes [2]. Montelukast Sodium (MON.Na) Fig. 1b, is chemically 1-[[[(R)-m-[(E)-2-[7-chloro-2-quinolyl] vinyl]-α-[o-(1-hydroxy-1-methyl ethyl) phenethyl] benzyl]thio]-methyl]cyclopropaneacetate [1], (MON.Na) is an antagonist of cysteinyl leukotriene receptor, on 20/2/1998 FDA approved the utilization of MON for chronic handling of asthma, preventing airway edema, smooth muscle contraction and enhanced secretion of thick, viscous mucus [3]. Literature showed several analytical methods for the estimation of each drug individually. Fexofenadine HCl was estimated individually by some analytical methods such as HPLC [4–6]—HPTLC [7]—spectrophotometry [8–11]—fluorimetry [12]—capillary electrophoresis [13]—potentiometry [14]. Similarly, Montelukast sodium (MON.Na) was determined using some analytical techniques such as HPLC [15–17], UV spectrophotometric [15, 18], capillary electrophoresis [19], Potentiometric [20, 21], and voltammetry [22]. The combination remedy of Fexofenadine with Montelukast sodium supplies enhanced effect by reducing the symptoms efficaciously [23], the determination of these drugs as combined dosage forms was limited by a few methods like HPLC [24–26], HPTLC [27] and derivative spectrophotometric methods [28, 29]. There was no previous electrochemical method for the determination of Fexofenadine HCl combined with Montelukast Na. The novelty in this presented work that we have created a new, accurate, sensitive, time and cost-saving potentiometric method for determination of Fexofenadine HCl and Montelukast sodium as combined form using pencil graphite electrodes depending on the difference in the active pH range for each sensor. Pencil graphite electrodes are considered a developed form of ion-selective electrodes. The advantages of these electrodes are the small size because there is no need for the internal filling solution, where we can use them in biological systems, their first response time, and long lifetime compared to those traditional ion-selective electrodes [30], in addition to the known advantages of the ion-selective electrodes such as being simple, accurate, economic, and saving time where there is no need for sample pretreatments such as extraction or filtration because of the ability of these electrodes to be used for analysing the turbid or colored solutions [31–35]. We have successfully applied this method for the determination of the combined dosage form without previous separation and that was our challenge.

Fexofenadine acts as a cation in that it makes up an ion pair with Molybdate anion, but montelukast acts as anion and makes up ion pair with the cationic reagent cobalt nitrate, therefore we can determine each drug separately without any interference of the other drug potential. The determination of Fexofenadine hydrochloride and Montelukast sodium in this presented work relies upon the construction of a pencil graphite electrode coated with a polymer film, which consists of polymer, plasticizer, and ion pair of previously mentioned drugs and reagents. The ion pairs are considered the active part in the electrode. The role of polymer is to provide mechanical support to other components of membrane film, which covers the graphite rod. The plasticizer gives an appropriate pliancy of the coating film. Among various types of ion-selective electrodes, pencil graphite electrode shows good conductivity, high sensitivity, small background current, and simple preparation [36]. The electrode’s potential arises from the contact of two layers, the coating membrane/drug solution layer and coating membrane/graphite layer. Thus, the cell potential is regarded as the potential difference between the two layers, and is calculated according to Nernstian equation.

$$ {\text{E }} = {\text{ E}}0 \, + { 2}.{3}0{3} = {\text{RT}}/{\text{ZF log }}\left[ {{\text{FEX}}} \right] $$

where; E: is the cell potential, E_0_: is the standard cell potential, R: is the universal gas constant^1^, T: is the temperature in Kelvins, Z: is the charge of the ion, F is the Faraday constant.

## Experimental

### Apparatus

Potentiometric measurements have been carried out using Radiometer analytical—ion check 10 pH/mv meter (CEDEX- France), all pH measurements have been carried out utilizing Crison pH meter model Glp21/EU (Spain), ultrasonic bath model Power Sonic 405 (Korea). All weights were taken by Sartorius balance model 2474 (Germany) its accuracy is ± 0.1 mg.

### Materials and chemicals

High pure Fexofenadine hydrochloride and Montelukast sodium were obtained by Sigma Aldrich, analytical grade ammonium molybdate, cobalt nitrate (BDH chemicals, England), high molecular weight PVC (SABC. KSA), tetrahydrofuran solvent (MERCK 99.5%), di- butyl phthalate (MERCK 99%).

### Standard drug solutions

#### FEX stock standard solution (1.00 × 10^–2^ mol L^−1^)

The FEX stock solution was prepared by dissolving accurate weight in bi-distilled water, and then the volume was made up to the mark into a 50-mL volumetric flask.

#### MON stock solution (1.00 × 10^–2^ mol L^−1^)

The MON stock solution was prepared by dissolving accurate weight in bi-distilled water, and then the volume was made up to the mark into a 50-mL volumetric flask.

#### Working solutions

A series of working solutions, their concentrations varying (1.00 × 10^–7^–1.00 × 10^−3^ mol L^−1^), were prepared by serial dilutions from the stock solutions using bi-distilled water.

### Procedure

#### Preparation of FEX.Mol ion pair

The ion pair of fexofenadine cation with molybdate anion was prepared by mixing 1 mmol of Fexofenadine hydrochloride with 1 mmol of molybdate ammonium. An off-white precipitate was formed, then the precipitate was filtered and washed several times by bi-distilled water. The conductivity of the filtrate was checked to be ≤ 2 µs/cm which confirmed the disposal of all obstructive ions [37].

### Preparation of MON.Co ion pair

The ion pair of Montelukast anion with cobalt cation was prepared by mixing of 1 mmol of Montelukast sodium with 2 mmol of cobalt nitrate. A pink precipitate was formed, then the precipitate was filtered and washed several times by bi-distilled water. The conductivity of the filtrate checked to be ≤ 2 µs/cm which confirmed the disposal of all obstructive ions [37].

### Fabrication of FEX pencil graphite coated electrode

The coating solution was prepared by mixing 0.45 g PVC with 0.9 g DBP, then 0.15 g of ion Pair (FEX.Mol) was added. All the components were dissolved in a small volume of THF. In this solution, a graphite rod was immersed several times to get a homogeneous layer of the coating material on the graphite rod. The coated graphite electrode was activated before the measurement of the potential, by dipping it in 1.00 × 10^–3^ mol/l FEX solution for 24 h [38].

### Fabrication of MON pencil graphite coated electrode

The coated solution was prepared by mixing 0.6 g PVC with 1.2 g DBP, then 0.2 g of ion Pair (MON.Co) was added. All the components were dissolved in a small volume of THF. In this solution, a graphite rod was immersed several times to get a homogeneous layer of the coating material on the graphite rod. The coated graphite electrode was activated before the measurement of the potential, by dipping it in 1.00 × 10^–3^ mol L^−1^ MON solution for 24 h [38].

### Fabrication of FEX&MON pencil graphite electrode (the combined electrode)

The preparation of this electrode was done by mixing 0.2 g of IP1 + 0.2 g of IP2 with 0.7 g PVC and 0.9 g DBP. All the components were dissolved in a small volume of THF. In this solution, a graphite rod was immersed several times to get a homogeneous layer of the coating material on the graphite rod. The coated graphite electrode was activated before the measurement of the potential, by dipping it in (1.00 × 10^–3^ mol L^−1^) FEX and MON solutions separately for 24 h in each solution.

### Direct potentiometric determination of Fexofenadine hydrochloride

A standard series of Fexofenadine hydrochloride (1.00 × 10^–7^–1.00 × 10^–2^) mol L^−1^ was prepared accurately. The potentiometric measurements were carried out using (1and 3) graphite coated electrodes in junction with Ag/AgCl reference electrode [37]. The potential produced by the proposed electrodes was recorded for each concentration to get the regression equations, which was used to determine this drug.

### Direct potentiometric determination of Montelukast sodium

A standard series of Montelukast sodium (1.00 × 10^–7^–1.00 × 10^–2^) mol L^−1^ was prepared accurately. The potentiometric measurements were carried out using the (1 and 3) graphite coated electrodes in junction with Ag/AgCl reference electrode [37]. The potential produced by the proposed electrodes was recorded for each concentration to get the regression equations, which was used to determine this drug.

### Optimization of experimental conditions

#### Effect of pH

The effect of pH on the potential response of the two sensors was studied over the pH ranges of [2–6] for Fexofenadine and [3–11] for Montelukast. This was obtained by adding diluted aliquots of (0.1 mol L^−1^) hydrochloric acid or sodium hydroxide solutions to the (1.00 × 10^–3^ and 1.00 × 10^–4^) mol L^−1^ drug solutions. The potential obtained at each pH value was recorded [39].

### Selectivity of the electrodes

The sensitivity of the constructed sensors was studied in the presence of some obstructive ions and excipients, which may exist with the drug material. The selectivity was studied using the matched potential method. In this method, the selectivity coefficient is characterized as the activity ratio of the essential and the interfering ion that shows the same potential change [39].

$$ {\text{K}}\, = \,\left( {\alpha^{\prime}_{{\text{A}}} {-}\alpha_{{\text{A}}} } \right) / \alpha_{{\text{B}}} $$

where; K is the selectivity coefficient, α'A is the activity of the primary ion, αA is the fixed activity of the primary ion, αB is the activity of interfering ion.

### Determination of FEX and MON in laboratory prepared mixtures

Different ratio mixtures of FEX and MON solutions were prepared. To do that, different volumes of the stocks solutions for both drugs were mixed to get a specific concentration of each drug which must be within the linearity range [40]. Each drug was determined using its proposed sensor in the presence of the other drug, depending on the effective pH range for each electrode.

### Preparation of test solutions

#### a. The determination of FEX.HCl in its pharmaceutical dosage form

For the determination of FEX.HCl in its pharmaceutical dosage form as a single drug, 20 tablets were finely powdered; exact weight proportionate to one tablet was taken, dissolved with bi-distilled water, and sonicated the solution in the ultrasonic bath for 5 min. Then the solution was filtered, an appropriate volume was taken from the filtrate and diluted with bi-distilled water in a 25 ml volumetric flask to get 1.00 × 10^–4^ mol L^−1^ of drug solution.

#### b. The determination of MON.Na in its pharmaceutical dosage form

For the determination of MON.Na in its pharmaceutical dosage form as a single drug, 20 tablets were finely powdered; exact weight proportionate to one tablet was taken, dissolved with bi-distilled water, and sonicate the solution in the ultrasonic bath for 15 min. Then the solution was filtered, an appropriate volume was taken from the filtrate and diluted with bi-distilled water in a 25 ml volumetric flask to get 1.00 × 10^–4^ mol L^−1^ of drug solution.

#### c. The determination of FEX& MON as a combination form

According to the common combination ratio of FEX&MON formulation, the binary mixture was prepared in ratio 12:1. precisely weighed (120 mg) FEX and (10 mg) MON then, common excipients that are used in the tablet formulation were added, the mixture was transferred to a 50 ml volumetric flask and diluted to the mark by bi-distilled water. For 20 min the solution was sonicated and filtered. From the filtrate, 10 ml was taken and diluted to 25 ml in volumetric flask by bi-distilled water to get the sample solution.

## Results and discussion

For several years great efforts have been devoted to the study of the Combined dosage forms, and that requires working in parallel to develop new analytical methods to analyse these combinations. The literatures in hand revealed that the determination of the combination of Fexofenadine and Montelukast were limited to HPLC and spectrophotometric methods, and there isn’t any previous analysis of both FEX and MON using potentiometric technique, which encourages us to propose new graphite sensors for the determination of this combination. The recovery values in Tables 3 and 4 indicate the accuracy and the specificity of the proposed method. The scientific novelty of the present work is that the used method is less expensive and less time consuming compared with other published HPLC, and spectrophotometric methods [41]. It also competes with the other methods in terms of the sensitivity and selectivity as shown in the results.

### Calibration of the electrodes

**Fig. 2:**
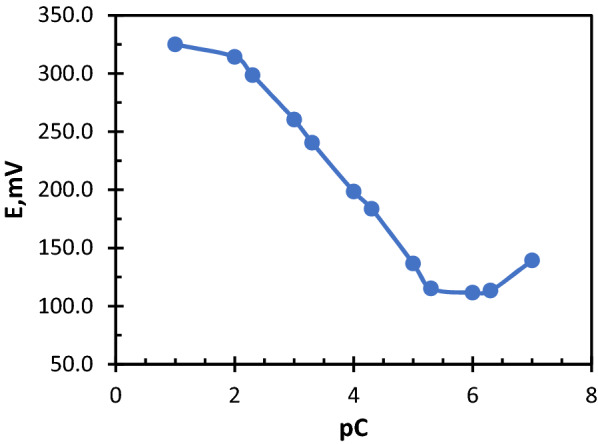
Potentiometric profile of FEX.Mol sensor

**Fig. 3:**
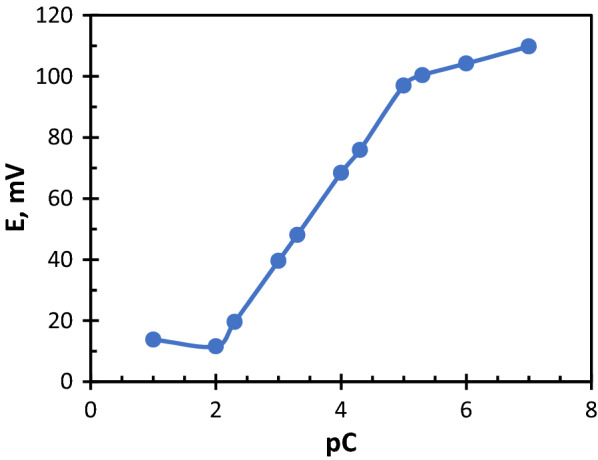
Potentiometric profile of MON.Co sensor

The constructed electrodes were dunked into a standard series solution of each drug; their concentration range (1.00 × ^10−7^–1.00 × 10^–1^) mol L^−1^, the potential of each solution was recorded, then a calibration graphs were plotted between the potential and the minus logarithm of drug concentration as shown in Figs. 2 and 3. The validations rules were applied according to ICH recommendations and the results are shown in Table 1. The sensors showed to be active for 69 days for FEX.Mol, and 45 days for MON.Co sensor. During these days, the slope of the regression equation was measured and found to be almost stable, but after this duration the slope was decreased obviously.

**Table 1:** Response characteristics and the validation data of the constructed sensors

Parameter	FEX.Mol	MON.Co	The combined sensor	
			FEX	MON
Slope ± SD (mV.decade^−1^)	− 59.23 ± 0.05	28.43 ± 0.09	− 59.05 ± 0.70	28.64 ± 0.22
Intercept (mV)	435.1	− 45.6	439.2	− 44.3
Correlation coefficient	0.9991	0.9998	0.9999	0.9998
Response time (seconds)	20	27	29	32
pH range	(2–4.5)	(5–9.5)	(2–4.5)	(5–9.5)
Linearity range (mol L^−1^)	(1.00 × 10^–2^–1.00 × 10^–5^)	(1.00 × 10^–2^–1.00 × 10^–5^)	(1.00 × 10^–2^–1.00 × 10^–5^)	(1.00 × 10^–2^–1.00 × 10^–5^)
Life time (days)	69	45	45	
Recovery ^a^ %	99.84 ± 0.51	100.92 ± 0.21	99.76 ± 0.50	100.55 ± 0.71
Repeatability ^b^	1.59	1.18	1.70	1.63
Reproducibility ^c^	1.73	0.29	1.91	1.99
Lod^d^ (M)	1.4 × 10^–8^	2.1 × 10^–8^	2.5 × 10^–8^	1.9 × 10^–8^
Loq (M)	4.3 × 10^–8^	6.3 × 10^–8^	7.6 × 10^–8^	5.9 × 10^–8^

^a^Average of three determinations

^b^Repeatability: the intraday precision (n = 3 × 3), average of three concentrations (5*10^–5^, 5*10^–4^ and 5*10^–3^ mol L^−1^) were repeated three times within the day

^c^Intermediate precision: the interday precision (n = 3 × 3), average of three concentrations

(5*10^–5^, 5*10^–4^ and 5*10^–3^ mol L^−1^) were repeated three times on two consecutive days

^d^Lod 3.3 SD of intercept/ slope, LOQ = 10*SD/ slope

### Effect of pH

**Fig. 4:**
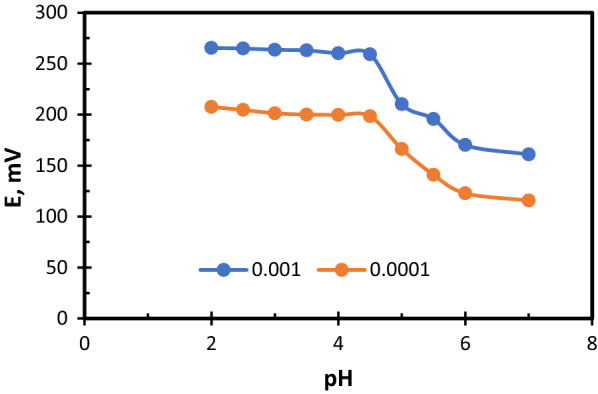
Effect of pH on potentiometric response for FEX.Mol sensor

**Fig. 5:**
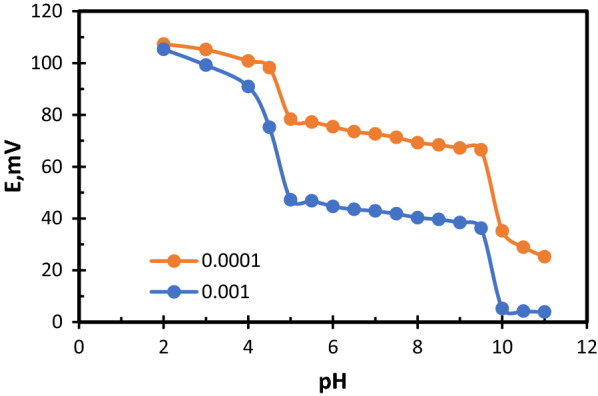
Effect of pH on potentiometric response for MON.co sensor

The effect of pH on the measured potential was studied. To do that, different Fexofenadine solutions, their pH values range (2–6), were prepared. The potential was measured for each solution using FEX.Mol graphite sensor. We found that the potential stays stable between pH range (2.5–4.5), at pH value more than 4.5, a noticed decrease in potential was found. For MON.Co sensor, different Montelukast solutions, their pH values range (3–11), were prepared. The potential was measured for each solution using MON.Co sensor. The effective pH range was found to be (5–9.5), at pH values less than 5, Montelukast drug participated, and more than 9.5, there was a decrease in the measured potential. It was found that there is no requirement for using any buffer, as buffers may involve some obtrusive substances, and because of the wide range of pH for both sensors (I and II). The obtained results are shown in Figs. 4 and 5.

### Selectivity of the constructed electrodes

The potential response of the proposed sensors in the presence of several related substances was studied, and the potentiometric selectivity coefficients were calculated to estimate the selectivity of the electrodes towards the primary drug ion (FEX) in case of sensor I and (MON) in case of sensor II, in the presence of the other drug ion and some obstructive ions which may exist in the drug solution. As shown in Table 2, the constructed electrodes show a good selectivity in the presence of the other drug which confirms the ability of determination of each drug in the combination dosage forms.

**Table 2:** Selectivity coefficients of the coated graphite constructed sensors

Interfering B	Sensor 1 (FEX.mol)	Sensor 2 (MON. co)	The combined sensor	
			FEX	MON
	K Fex,B	K Mon,B	K Fex,B	K Mon,B
CaCl2	4.9*10–3	3.2*10–2	4.9*10–3	3.4*10–2
KCl	1.3*10–3	4.6*10–3	1.7*10–3	4.6*10–3
NH4Cl	6.1*10–3	2.1*10–2	6.8*10–3	2.3*10–2
NaCl	1.3*10–3	3.0*10–3	1.5*10–3	3.5*10–3
dextrose	7.4*10–3	6.1*10–3	7.7*10–3	6.4*10–3
Mg stearate	2.4*10–3	8.7*10–3	2.7*10–3	8.9*10–3
Avicel	6.5*10–3	5.5*10–3	6.7*10–3	5.6*10–3
FEX	–	3.8*10–3	–	4.2*10–3
MON	5.5*10–2	–	5.6*10–2	

### Potentiometric determination of laboratory prepared mixtures containing different ratios of FEX and MON

The potential of the laboratory prepared mixtures containing different ratios of FEX and MON was measured, and the results showed that the proposed sensors FEX.Mol and the combined sensor can be effectively used for selective determination of FEX in the presence of MON, and the proposed sensor MON.Co and the combined sensor can be successfully used for selective determination of MON in the existence of FEX without a need for any previous separation, just we need to adjust the pH of each solution within the effective pH range for each electrode. The results are summarized in Table 3.

**Table 3:** Potentiometric determination of laboratory prepared mixtures containing various ratios of FEX and MON

Ratio		Recovery %			
FEX	MON	FEX		MON	
		Sensor 1	Sensor 3	Sensor 2	Sensor 3
1	1	98.40	98.22	99.31	98.89
5	1	97.27	97.13	99.97	99.20
10	1	100.92	100.52	101.62	101.12
12	1	101.16	100.99	98.40	98.14
1	12	97.72	97.56	97.58	97.33
Mean ± SD		99.09 ± 1.82	98.88 ± 1.76	99 ± 1.84	98.94 ± 1.42

### Potentiometric determination of the sample solution

The prepared sensors in conjunction with the double junction Ag/AgCl reference electrode were soaked separately in the sample solution after the adjusting of pH value of the sample solution within the effective pH range of each electrode. The resulting potential was recorded, the corresponding concentration was calculated from the regression equations for each sensor. We have successfully determined each of Fexofenadine and Montelukast drugs in their combination form without any need for any previous separation. The excipients, which were added, did not influence the potential response. That approves the ability of the developed method for the determination of Fexofenadine and Montelukast in their binary dosage form. The results were compared with the results obtained by reference UV spectroscopic methods [8, 42], the statistical tests show that there is no significant difference in the results by applying the two methods as shown in the Table 4.

**Table 4:** Determination of FEX and MON in pharmaceutical preparations using the proposed method and reference methods

Commercial Name	Composition	Amount found, mg^a^	R% ± SD	t-value^b^	F-value^c^
Sensor 1 FEX.Mol					
Fexofenadine	Fexofenadine 120 mg	119.37	99.47 ± 1.16	1.06	3.53
Azmalir	Montelukast 10 mg	–	–	–	–
Combination form	Fexofenadine 120 mg	119.27	99.39 ± 0.87	1.96	3.39
	Montelukast 10 mg	–	–	–	–
Sensor 2 MON.Co					
Fexofenadine	Fexofenadine 120 mg	–	–	–	–
Azmalir	Montelukast 10 mg	10.05	100.5 ± 1.74	4.07	1.66
Combination form	Fexofenadine 120 mg	–	–	–	–
	Montelukast 10 mg	9.97	99.71 ± 1.61	2.82	3.20
Sensor 3 FEX.MOl + MON.Co					
Fexofenadine	Fexofenadine 120 mg	119.53	100.39 ± 0.78	2.26	3.46
Azmalir	Montelukast 10 mg	10.12	98.80 ± 1.20	2.77	1.77
Combination form	Fexofenadine 120 mg	120.83	100.69 ± 0.69	2.13	2.95
	Montelukast 10 mg	9.89	98.88 ± 1.34	2.22	3.30

^a^Average of 3 replicates

^b^t critical 4.302 (0.05)

^c^f critical 19 (0.05), n = 3

## Conclusion

This research was the first electrochemical method for the determination of Fexofenadine hydrochloride and Montelukast sodium combination. This paper has clearly shown that the designed graphite sensors seem to give important results in terms of detection limit, long life-time, and selectivity. Thus, it could compete with the many sophisticated methods which were reported to determine this combination. The validation outcomes showed that the constructed method was accurate, precise, and sensitive for the determination of each drug as pure form, laboratory prepared mixtures, and pharmaceutical formulation without any separation steps. Based on the results, it can be concluded that the coated graphite electrodes offered a powerful and versatile analytical technique as well as a large linear dynamic range, with relatively low-cost instrumentation for the determination of drugs, so we suggest using this type of electrode in drug analysis.

## Data Availability

The datasets used and analysed during the current study are available from the corresponding author on reasonable request.

## Abbreviations

FEX: Fexofenadine hydrochloride
MON: Montelukast sodium
FDA: Food and drug administration
Mol: Ammonium molybdate
Co: Cobalt nitrate
ICH: The International Council for Harmonization of Technical Requirements for Pharmaceuticals for Human Use
PVC: Poly venyl chloride
DBP: Di-butyl phthalate
THF: Tetrahydrofuran
HPLC: High performance liquid chromatography
HPTLC: High performance thin layer chromatography
